# Smoking During Pregnancy and Its Association with Maternal Health Among Women of Reproductive Age in Alabama: An Analysis Using the Three Delays Model and PRAMS Phase 8 Data

**DOI:** 10.3390/healthcare13111277

**Published:** 2025-05-28

**Authors:** John Kwame Duah

**Affiliations:** College of Liberal Arts, Department of Political Science, Health Services Administration Program, Auburn University, Auburn, AL 36849, USA; jkd0046@auburn.edu; Tel.: +1-334-844-6234

**Keywords:** PRAMS, Three Delays Model, maternal health, smoking, prenatal care, barriers to care, hierarchical clustering analysis, Alabama

## Abstract

Background/Objectives: Smoking during pregnancy endangers maternal and infant health outcomes, posing significant risks such as preterm birth and developmental complications. Applying the Three Delays Framework, this study examines how smoking behaviors impact maternal health in Alabama, focusing on barriers to prenatal care. Methods: This cross-sectional study utilized PRAMS Phase 8 data collected from 2016 to 2021, comprising 533 observations from the Alabama Department of Public Health. Prenatal care barriers were categorized into three domains: seeking, reaching, and receiving care. Analyses included descriptive statistics, hierarchical clustering of barrier items, derivation of an informational barrier latent score using item-response theory (θ_info), and multivariable logistic regression adjusted for age, race/ethnicity, income, and BMI. Results: Multivariable logistic regression analysis revealed that informational barriers (OR = 2.42, *p* < 0.001) and system-level barriers (OR = 1.77, *p* = 0.047) were significant predictors of adverse maternal health outcomes. In the baseline model, which included sociodemographic covariates, prenatal smoking was not significantly associated with poor maternal health (OR = 0.83, 95% CI [0.58, 1.17]). After adjusting for informational, structural, and system barriers, the association further attenuated and remained non-significant (adjusted OR = 0.72, 95% CI [0.50, 1.03]). Conclusions: While smoking during pregnancy remains a modifiable health behavior, informational and system barriers emerged as the more immediate determinants of poor maternal outcomes in this cohort. Addressing these barriers through targeted prenatal education initiatives and system-level improvements can enhance care utilization and improve maternal and infant health outcomes, even among pregnant smokers.

## 1. Introduction

Smoking during pregnancy poses substantial risks to maternal and neonatal health, contributing to adverse outcomes such as stillbirth, preterm birth, birth defects, and low birth weight [1]. Despite efforts to address this issue, maternal mortality and morbidity rates in Alabama continue to exceed national benchmarks, driven in part by high smoking prevalence among reproductive-age women and barriers to accessing prenatal care [2]. According to the Alabama Maternal Mortality Review Committee, pregnancy-related mortality and near-miss events remain alarmingly high despite declines at the national level, highlighting the need for a localized understanding of delayed or suboptimal care [3]. More crucially, regional studies have documented significant barriers impeding timely prenatal care utilization, such as transportation deficits, appointment delays, and stigma-driven informational gaps [2,3,4]. These barriers collectively exacerbate the challenges faced by pregnant women, particularly smokers, in accessing essential healthcare services.

Historically, U.S. policy has alternated between punitive measures, such as criminalizing prenatal substance use, and public health-focused interventions aimed at providing support [5]. In states like Alabama, the chemical endangerment law categorizes prenatal substance use as child endangerment, deterring some women from seeking care due to fear of legal repercussions [6,7]. In addition, the burden of disease and disability associated with smoking during pregnancy is continually changing. Therefore, it is essential to prioritize and update our understanding of the structural and social factors that contribute to this public health challenge to improve outcomes for those affected by this issue. Furthermore, long-term maternal outcomes, such as postpartum morbidity and mental health challenges, remain insufficiently addressed, particularly in settings where substance use stigma is prevalent [8,9].

While significant efforts have been made over the past decades to lessen the risks associated with smoking during pregnancy among women of reproductive age, substantial gaps remain in contextualizing the psychosocial factors that contribute to this problem and how to galvanize community action to address this ongoing health issue [10]. Interestingly, past research has shown that psychosocial and behavioral interventions, like contingency management, motivational support, and cognitive behavioral therapies, are crucial to addressing substance use during pregnancy [11]. Notwithstanding, establishing and implementing innovative, sustained strategies that can help identify at-risk pregnant women early and connect them to treatment remains a significant challenge in resource-constrained settings, underscoring the critical importance of information-seeking during the prenatal period.

Moreover, geographic factors and cultural nuances play a critical role in shaping the burden and disability associated with smoking during pregnancy, varying significantly across diverse care settings. The prevailing perspective suggests that culture significantly shapes behavior and communication, with its intricate nuances influencing beliefs and perceptions regarding substance and alcohol use [12]. This insight underscores the importance of uncovering the multifaceted dynamics and interconnected factors that drive pregnant women affected by substance use to seek, access, and successfully receive care.

Against this backdrop, the Three Delays Model provides a structured framework to categorize and address multifaceted barriers at critical points in the prenatal care continuum [13]. This framework identifies delays in seeking care, reaching healthcare facilities, and receiving adequate care, allowing for a comprehensive understanding of the obstacles that impact maternal and infant health outcomes. Delays in seeking care are often related to various factors, including informational barriers, insufficient awareness about pregnancy signs, and hesitancy in recognizing the importance of prenatal care. Systemic factors, such as transportation difficulties or clinic access issues, contribute to delays in reaching care [2,9], and delays in receiving care reflect structural barriers, such as long wait times and unsupportive clinic environments.

In the context of Alabama, these barriers are amplified by stigma and systemic challenges, making it crucial to employ frameworks like the Three Delays Model to improve maternal health outcomes. This study applies the Three Delays Model to assess whether informational, structural, and system barriers confound and thereby attenuate the crude association between prenatal smoking and maternal health.

Accordingly, the study tests two hypotheses:

1. Higher levels of prenatal care barriers, especially informational and system domains, are associated with poorer self-reported maternal health;
2. Adjusting for these barrier domains will substantially weaken or eliminate the crude association between prenatal smoking and poor maternal health.

### Conceptual Framework and Study Aims

The Three Delays Model lists or delineates three stages at which barriers can impede prenatal care [13]:

I. Delay 1 (Decision to Seek Care): This domain or phase focuses on informational/sociocultural barriers. It touches on issues like a lack of pregnancy awareness, fear of legal repercussions, or stigma that discourages the initial decision to seek care;
II. Delay 2 (Reaching Care): This phase or domain focuses on structural barriers, including transportation problems, work/school conflicts, childcare responsibilities, or out-of-pocket costs that obstruct travel to a facility;
III. Delay 3 (Receiving Care): This phase or domain highlights system barriers, including appointment availability, insurance restrictions, or provider delays that hinder the timely receipt of services once a facility is reached.

In this study, prenatal smoking serves as the exposure, while self-reported maternal health is the outcome. The three delay-domain barriers are evaluated as potential confounders that may attenuate the crude smoking health association. Because some barriers, such as stigma-related informational deficits, can both influence smoking behavior and directly impact health, treating them as confounders aligns with current causal-inference guidance [14,15]. Accordingly, the study addresses two aims:

1. Estimate the independent associations between each delay-domain barrier and maternal health status;
2. Determine whether adjusting for informational, structural, and system barriers reduces or eliminates the crude association between prenatal smoking and poor maternal health, after controlling for age, race/ethnicity, income, and BMI.

## 2. Materials and Methods

This cross-sectional study utilized secondary de-identified data from the Pregnancy Risk Assessment Monitoring System (PRAMS) Phase 8 survey (2016–2021; N = 533) provided by the Alabama Department of Public Health. PRAMS is administered to women who have recently delivered a live infant and captures maternal behaviors and attitudes during pregnancy and the early postpartum period, typically 2–4 months after birth. The population of interest comprised women who experienced live births in Alabama from 2016 to 2021. The initial PRAMS Phase 8 data set used for analysis comprised 596 observations, deliberately selected based on key variables essential to addressing the research question, study aims, and the relevance of the Three Delays Model or framework. A total of 63 records missing any key variables were excluded, with listwise deletion applied across all analyses.

### 2.1. Measures

The study variables were conceptualized using the Three Delays Model. The outcome, maternal health status, was derived from PRAMS Qn22 items and dichotomized as good vs. poor based on self-report. Although PRAMS captures multiple substances, cigarette items showed the highest response reliability; prenatal smoking was measured with Qn42_CigaretteCategory (“In the last 3 months of your pregnancy, how many cigarettes on an average day?”) and recoded 0 = non-smoker, 1 = any smoking.

Delay-domain confounders

- Delay 1—Informational barriers: latent trait θ_info from IRT modelling of Qn21i (*I didn’t know I was pregnant*), Qn21j (*I didn’t want anyone else to know*), and Qn21k (*I didn’t want prenatal care*), capturing stigma-related knowledge deficits;
- Delay 2—Structural barriers: sum of Qn21b (financial constraints) + Qn21f (work/school conflicts) + Qn21h (no childcare);
- Delay 3—System barriers: sum of Qn21a (appointment availability) + Qn21d (insurance/plan delays).

The covariates were age group (15–19, 20–24, 25–29, 30–34, 35–39, and ≥40 y), race/ethnicity (Non-Hispanic White, Non-Hispanic Black, and Hispanic), household income (Qn79 categories), and BMI category (underweight, healthy, and overweight, obese). Table 1 provides operational definitions, coding schemes, and the Qn21 mapping to delay domains; Appendix A (Table A2) lists full PRAMS wording for each barrier item.

Factors that influence substance use and smoking during pregnancy among women of reproductive age are multifaceted [16,17,18]. Addressing this intractable public health crisis requires the application of an appropriate theoretical framework coupled with a steadfast commitment to sustainable resource allocation. This study employs the Three Delays Framework to provide critical insights into the multifaceted challenges women experience when seeking prenatal care services during pregnancy [13]. By capturing the interplay of individual, community, and health system factors, this framework offers a structured approach to identifying modifiable barriers and their linkages, offering opportunities for effective policy realignment and addressing implementation gaps.

### 2.2. Statistical Analysis

Descriptive statistics were generated to summarize sample characteristics and variable distributions. Multidimensional scaling (MDS) and hierarchical clustering techniques were used to assess the grouping of prenatal care barrier items. Internal consistency was evaluated using Cronbach’s alpha (α) and McDonald’s omega (ω). Despite the low reliability estimates (ω = 0.11, 0.07, 0.14), the composites were retained because the items represent theoretically coherent domains within the Three Delays framework, and the study is intentionally exploratory. Item response theory (IRT) modeling was then applied to derive the latent informational barrier trait (θ_info), accounting for item difficulty and discrimination.

Age categories were collapsed into three groups (15–24, 25–34, and ≥35 years), while household income was categorized as ≤USD 20k, USD 20–40k, and >USD 40k to ensure adequate cell sizes. Multivariable logistic regression was the sole inferential technique employed. The baseline model included smoking status and sociodemographic covariates (age band, race/ethnicity, income band, and BMI). A second model incorporated informational, structural, and system barriers as confounders. Odds ratios (ORs) with 95% confidence intervals (CIs) were reported, and model fit was assessed using the Hosmer–Lemeshow test and variance-inflation diagnostics, with no multicollinearity detected.

The adjusted model was specified as follows:

logit[P(Y = poor)] = β0 + β1(smoker_status) + β2(structural_barriers) + β3(system_barriers) + β4(θ_info) + β5(AgeBand3) + β6(Race/Ethnicity) + β7(IncBand3) + β8(BMI_Category)

All analyses were conducted in R 4.4.2 using the following packages: dplyr, tidyr, psych, and broom.

## 3. Results

The initial PRAMS Phase 8 data set comprised 596 observations. After excluding 63 records with missing key variables (listwise deletion), 533 remained for analysis.

### 3.1. Sample Characteristics

Table 2 displays frequencies and percentages for all key study variables (maternal health status, smoking status, income, BMI, age group, and race/ethnicity), except for the Qn21 barrier item frequencies, which are moved to Table A1 for ease of reading. For example, 53.8% of respondents were classified as having good maternal health, while 46.2% had poor maternal health. In addition, 60.2% were categorized as non-smokers, and 39.8% were smokers.

### 3.2. IRT-Derived Informational Barriers

**Figure 1:**
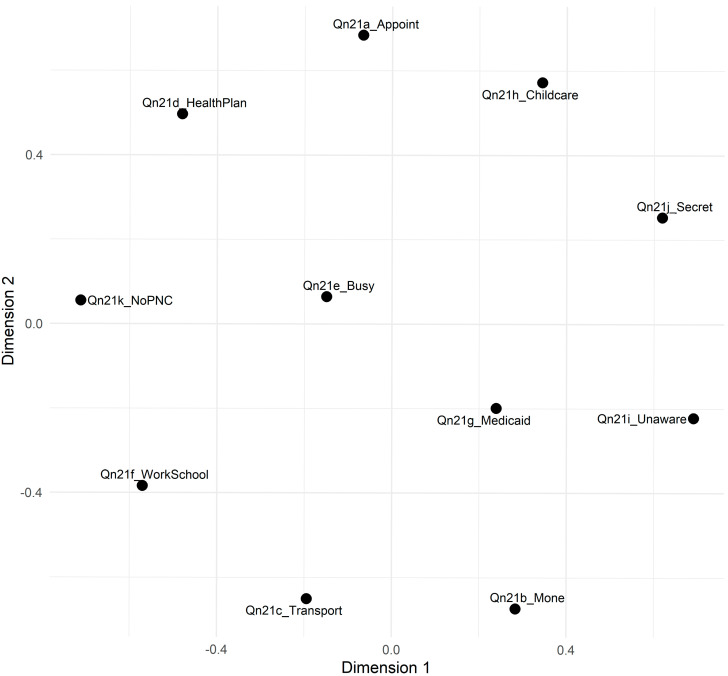
Multidimensional scaling of Qn21 items. Note: Distances represent the dissimilarity among items (-1|correlation|). Items closer together in the two-dimensional space share stronger correlations, suggesting they may reflect similar underlying barriers to prenatal care.

Figure 1 shows the two-dimensional MDS plot, where items cluster in patterns suggestive of distinct barrier domains. Items associated with financial or scheduling constraints, like *I didn’t have enough money or insurance to pay for my visit* (Qn21b) and *I couldn’t get an appointment* (Qn21a), appeared in one region, and items reflecting knowledge or secrecy barriers, like *I didn’t know that I was pregnant* (Qn21i), and *I didn’t want anyone else to know I was pregnant* (Qn21j), appeared in another. These patterns provide initial evidence that these items may capture multiple underlying dimensions of prenatal care barriers, aligning partially with the Three Delays Model. For a full list of the Qn21 items and their corresponding barrier domains, please refer to Table A2 (Appendix A).

To assess the relationship between different types of delays (seeking, reaching, and receiving care) and maternal health status, bivariate analyses were conducted using chi-square tests. The results indicated that the Delay Seeking factor was significantly associated with maternal health status (χ^2^(3) = 23.11, *p* < 0.001). In contrast, the Delay Reaching (χ^2^(1) = 2.59, *p* = 0.462) and Delay Receiving (χ^2^(1) = 4.42, *p* = 0.229) factors showed no significant associations. These findings suggest that, at the bivariate level, variations in Delay Seeking are the only factor significantly linked to differences in maternal health outcomes.

Expanding on these bivariate findings, it is noteworthy that although the Delay in Seeking barrier showed a significant bivariate association with maternal outcomes (χ^2^(3) = 23.11, *p* < 0.001), this effect was not retained in the multivariable models, suggesting that its impact is confounded or mediated by other barriers.

### 3.3. Multivariable Logistic Regression

Baseline results, which included smoking status and sociodemographic covariates, showed no significant association between prenatal smoking and poor maternal health (OR = 0.83, 95% CI [0.58, 1.17]). After adjusting for informational, structural, and system barriers as confounders, the association further attenuated and remained non-significant (adjusted OR = 0.72, 95% CI [0.50, 1.03]). Informational barriers (OR = 2.42, *p* < 0.001) and system-level barriers (OR = 1.77, *p* = 0.047) were the only factors that significantly increased the odds of poor maternal health, whereas structural barriers were not statistically significant. Model calibration was adequate (Hosmer–Lemeshow *p* = 0.61), and variance-inflation factors were ≤1.7, indicating no multicollinearity. Table 3 provides the full model estimates, while the baseline versus adjusted smoking odds ratio is listed in Appendix B, Table A3.

As shown in Table 3, after controlling for sociodemographic factors and the three delay domains, informational and system barriers, rather than smoking status, emerge as the strongest determinants of poor maternal health.

### 3.4. Hierarchical Clustering Analysis

**Figure 2:**
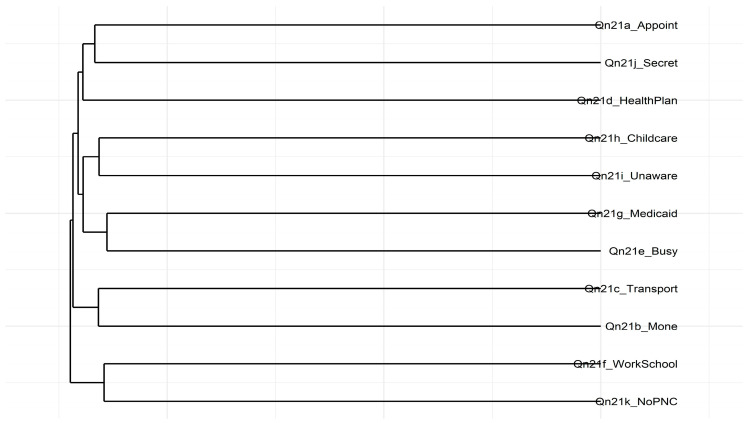
Hierarchical clustering dendrogram of Qn21 items. Note: The dendrogram was created using an average linkage method and a dissimilarity matrix derived from 1 − ∣r∣. Items that fuse at lower heights share stronger correlations, showing they measure similar or overlapping barriers to prenatal care.

A hierarchical clustering analysis was performed to identify distinct groups of prenatal care barriers. This method shows how specific barriers cluster together, offering insights into their relationships and guiding targeted perinatal interventions. Figure 2 depicts the hierarchical clustering dendrogram for the Qn21 items, which were hypothesized as representing different domains of prenatal care barriers. Several notable clusters emerged.

For instance, *I didn’t have enough money or insurance to pay for my visit* (Qn21b), and *I couldn’t take time off from work or school* (Qn21f) are grouped, showing a potential overlap in financial and work-related constraints. Similarly, *I didn’t have my Medicaid card* (Qn21g), *I had too many other things going on* (Qn21e), and *I didn’t know that I was pregnant* (Qn21i) formed another cluster, revealing a set of logistical or knowledge-related barriers. Conversely, *I didn’t want anyone else to know I was pregnant* (Qn21j), and *I didn’t want prenatal care* (Qn21k) appeared as a distinct pair, potentially capturing personal or privacy concerns associated with pregnant women not seeking prenatal care.

These groupings largely align with the patterns observed in the Multidimensional Scaling (MDS) analysis shown in Figure 1. However, the dendrogram does not wholly separate the items into three discrete clusters corresponding to the Three Delays Model. Instead, the results suggest that some barriers, like money and work constraints, may cut across multiple theoretical domains. Composite scores for each cluster were created to understand their relationship to maternal health outcomes. Further steps involve refining these item groupings and examining how they relate to maternal health outcomes.

### 3.5. Item Characteristics Curves

**Figure 3:**
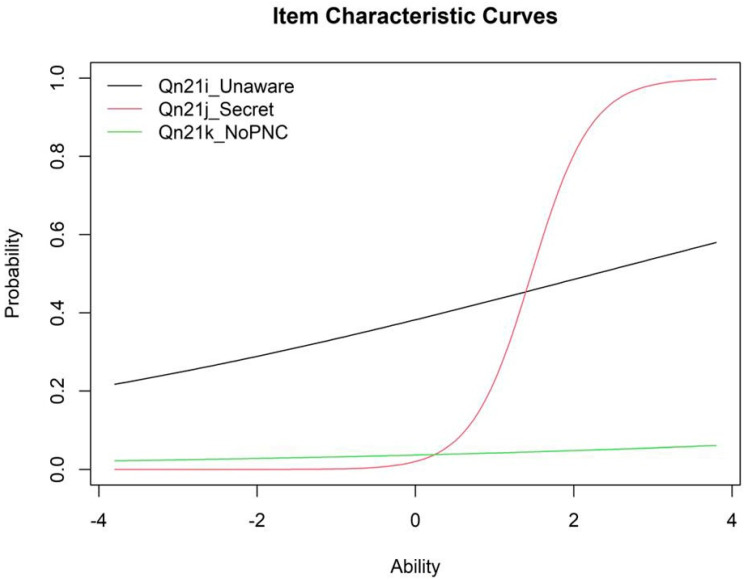
Item characteristic curves for informational barriers.

Figure 3 illustrates the item characteristic curves (ICCs) for the informational barriers’ items, underscoring their discriminative properties under a two-parameter logistic (2PL) IRT model. For instance, *I didn’t know that I was pregnant* (Qn21i) portrays a relatively flat slope, indicating low discrimination. In contrast, *I didn’t want anyone else to know I was pregnant* (Qn21j) shows a sharply declining curve, consistent with its strong discrimination and high negative loading, and *I didn’t want prenatal care* (Qn21k) demonstrates a steep slope, reflecting moderate-to-high discrimination.

These patterns show that *I didn’t want anyone else to know I was pregnant* (Qn21j) and I didn’t want prenatal care (Qn21k) are particularly effective in differentiating respondents along the latent trait of informational barriers, while *I didn’t know that I was pregnant* (Qn21i) contributes less to differentiation. Despite the limited discrimination of *I didn’t know that I was pregnant* (Qn21i), the items collectively capture a meaningful latent construct of informational barriers to prenatal care.

The ICCs visually highlight how each item differentiates across levels of the latent trait, further emphasizing the critical role of informational barriers in understanding maternal health outcomes. Although *I didn’t know that I was pregnant* (Qn21i) demonstrated low discrimination, it was retained to preserve theoretical completeness. Future studies may consider excluding this item.

## 4. Discussion

This cross-sectional study is the first to extend the Three Delays Model to prenatal smoking, identifying system and informational barriers, rather than structural constraints or smoking itself, as the primary factors associated with poor maternal health. Fear of mandatory reporting and its related stigma likely exacerbate Delay 1, discouraging pregnant smokers from seeking timely care [6,7,19]. Although the Three Delays Model is widely applied in maternal health research [13,20,21], its use in examining smoking during pregnancy remains limited. The study findings indicate that informational and system barriers independently predict adverse maternal outcomes and substantially attenuate the crude smoking health association, emphasizing the need for tailored educational campaigns that enhance awareness and navigation of prenatal services among high-risk populations.

To align with the Three Delays framework, prenatal care barriers were categorized into structural, informational, and system domains. Consistent with previous PRAMS analyses in Alabama [2], Georgia [3], and Mississippi [4], as well as national maternal health reports [22,23,24,25,26,27,28], informational and system barriers remained significant even after adjusting for age, race/ethnicity, income, and BMI. These barriers align with broader frameworks addressing substance use during pregnancy, such as contextual models of opioid crises [22]. Additionally, polysubstance use, combining smoking with alcohol or opioids, worsens prenatal health disparities [23], reinforcing the importance of maternal healthcare strategies designed to address worsening outcomes [24,25]. Global maternal health guidelines stress the need for equitable access to care, highlighting targeted prenatal outreach for high-risk populations [26].

Since the barrier domains act as confounders rather than mediators in the adjusted multivariable logistic regression model, the exploratory mediation and SEM outputs remain available in Appendix B (Table A3 and Table A4) for readers interested in alternative causal specifications. However, these supplementary analyses do not alter the primary conclusion that informational and system barriers are the predominant contributors to poor maternal health in this cohort. Structural equation modeling (SEM) was employed to assess mediation pathways, following methodological best practices for response shift detection and item response theory applications [27,28]. Findings from Tennessee PRAMS further reinforce these patterns, highlighting persistent disparities in maternal health access [29]. These results suggest that interventions addressing informational deficits, such as culturally tailored prenatal education modules, and system frictions, including appointment availability and insurance navigation, may yield greater improvements in maternal health than smoking-cessation messaging alone.

### 4.1. Implications for Theory and Practice

The study findings highlight the value of the Three Delays Model for analyzing barriers to prenatal care and their influence on maternal outcomes. System and informational barriers emerged as the most vital obstacles for pregnant smokers: inadequate appointment availability, insurance hurdles, and limited knowledge about prenatal services compound the challenges of navigating care.

Low maternal health literacy has long been linked to sub-optimal maternal and child health outcomes, including reduced access to essential services [30]. Likewise, language barriers and health communication anxiety among linguistic minorities can exacerbate disparities [31], while cultural norms and health-seeking behaviors shape service use in rural or diverse communities [32].

Importantly, disparities in postpartum care reinforce the need for equitable approaches. Interrante et al. [33] showed that race, geography, and insurance type affect receipt of recommended postpartum visits, underscoring the interconnected social and structural determinants of health. Shang et al. [9] demonstrated that early, targeted postpartum interventions reduce depression and anxiety among women with pregnancy complications, and Pacho et al. [34] found that substance use during pregnancy increases the risk of postpartum depression. Culturally tailored health education materials have been shown to improve patient engagement and health outcomes, especially among underserved populations [35].

Practical recommendations include establishing integrated antenatal substance counselling clinics and adopting a “no wrong door” referral system so that patients receive needed services regardless of entry point. Routine postpartum follow-ups are also essential to maintain continuity of care. In Alabama, interventions should prioritize easy-to-understand, culturally tailored prenatal care information and community support referrals, complemented by case management services to sustain health gains. Addressing systemic issues, such as appointment scheduling, transportation, and insurance barriers, should be a policy priority. Investments in telehealth and mobile clinics could further reduce geographic inequities.

Because informational and system barriers confound the smoking health association, tackling these domains may yield substantial improvements in maternal health beyond smoking cessation messaging alone.

### 4.2. Study Limitations

This study has several limitations that merit consideration. First, it focuses exclusively on smoking as a substance use behavior. This tobacco-only focus may overlook broader substance use patterns, such as alcohol or opioid use, that also influence maternal health outcomes. Future PRAMS modules should expand their scope to capture the full spectrum of substance use during pregnancy.

Second, the study findings are specific to Alabama and may not generalize to other states or regions. PRAMS incorporates state-specific core questions and sampling methods, which may limit cross-state comparability. Notwithstanding, this geographic specificity is also a strength, as it provides actionable insights for policymakers and practitioners in Alabama’s unique demographic and healthcare context.

Third, several additional sociodemographic and reproductive variables like maternal education level, marital status, parity (number of children), and pregnancy intendedness, align with the Three Delays framework as potential influences on the decision to seek care (Delay 1) or the ability to reach care (Delay 2). For instance, maternal education shapes health literacy and recognition of prenatal needs, and marital status and household structure provide social support for transportation and childcare. Parity affects caregiving responsibilities, and pregnancy intendedness reflects motivation to engage in care.

However, a substantial portion of the complete-case sample had missing information on one or more of these variables, and including them in multivariable models would have markedly reduced the analytic sample and risked biased estimates. Although conceptually relevant, these factors were excluded to preserve analytic validity. Future research with more complete data should examine their specific roles within the prenatal care continuum.

Fourth, the cross-sectional design precludes causal inferences, and associations observed cannot establish temporal ordering or definitively confirm mediation versus confounding. Fifth, some composite measures exhibited low internal consistency, requiring advanced techniques such as item response theory (IRT) to derive latent traits. While IRT addresses reliability concerns, it may obscure item-level nuances. Qualitative studies or longitudinal designs could complement these findings by providing richer, context-specific insights into prenatal care barriers and their impact on maternal health.

## 5. Conclusions

In this cross-sectional analysis of PRAMS Phase 8 data from Alabama, the Three Delays Model was applied to examine how barriers to prenatal care influence maternal health among pregnant smokers. After adjusting for age, race/ethnicity, income, and BMI, smoking status was not significantly associated with poor maternal health (adjusted OR = 0.72, 95% CI [0.50, 1.03]). Instead, informational barriers (OR = 2.42, *p* < 0.001) and system-level barriers (OR = 1.77, *p* = 0.047) emerged as the strongest predictors.

These findings underscore that, although smoking remains a vital public health target, the most immediate opportunities for improving maternal health lie in addressing modifiable obstacles to care, such as increasing awareness of prenatal services and streamlining appointment and insurance processes. Mediation and SEM results have been moved to Appendix B to ensure transparency while maintaining focus on the primary message that enhancing prenatal care utilization through targeted education campaigns and system-level reforms may yield greater improvements in maternal health than focusing exclusively on smoking cessation.

Future research should employ longitudinal designs, incorporate a broader set of sociodemographic and psychosocial covariates, and evaluate barrier-reduction strategies across diverse settings to confirm and extend these findings.

## Figures and Tables

**Table 1:** Operational definitions and measurement of study variables.

Variable	Operational Definition	Coding/Measurement	Data Source
Maternal Health Status	Refers to overall maternal health and operationalized as good vs. poor	The composite variable from Qn22 items is coded as 1 = good and 2 = poor	ADPH PRAMS Phase 8 data set (2016–2021)
Smoking Status	This refers to pregnant women’s smoking behavior during pregnancy	The binary variable from Qn42_CigaretteCategory is coded as 0 = non-smoker and 1 = smoker	ADPH PRAMS Phase 8 data set (2016–2021)
Structural Barriers	This refers to the financial constraints and work/school conflicts experienced when accessing care	The composite score of Qn21b_Money and Qn21f_WorkSchool	ADPH PRAMS Phase 8 data set (2016–2021)
Informational Barriers	This refers to deficits in awareness or information regarding prenatal care	This is the IRT-derived latent trait (theta_info) from Qn21i_Unaware, Qn21j_Secret, and Qn21k_NoPNC	ADPH PRAMS Phase 8 data set (2016–2021)
System Barriers	This refers to barriers related to navigating the healthcare system	This is the composite score of Qn21a_Appoint and Qn21d_HealthPlan	ADPH PRAMS Phase 8 data set (2016–2021)
Age Group	This refers to the age category of the respondent	This is a categorical variable, grouped as 15–19, 20–24, and above	ADPH PRAMS Phase 8 data set (2016–2021)
Race/Ethnicity	This refers to the self-reported race and ethnicity of respondents	This is a categorical variable coded as 1 = Non-Hispanic White, 2 = Non-Hispanic Black, and 3 = Hispanic	ADPH PRAMS Phase 8 data set (2016–2021)
Qn79_Income Category	This refers to the household income level reported by respondents (Qn79 items)	This is a categorical variable, with income categories ranging from USD 0 to USD 16,000, USD 16,001 to USD 20,000, USD 20,001 to USD 24,000, USD 24,001 to USD 28,000, and above	ADPH PRAMS Phase 8 data set (2016–2021)
BMI Category	This refers to the body mass index (BMI) classification provided by the Alabama Department of Public Health (ADPH)	This is a categorical variable, coded as 1 = Healthy Weight, 2 = Overweight, and 3 = Obesity	ADPH PRAMS Phase 8 data set (2016–2021)

Note: The maternal health outcome is derived from Qn22 items, while substance use is based on Qn42_CigaretteCategory. Prenatal care barriers are grouped into structural, informational, and system domains in accordance with the Three Delays Model.

**Table 2:** Sociodemographic and health characteristics of participants (Complete-Case Analytic Sample, N = 533).

Variable	Category	n	%
Maternal health status	Good	287	53.8
	Poor	246	46.2
Smoking status	Non-smoker	321	60.2
	Smoker	212	39.8
Household income	≤USD 20,000	188	35.3
	USD 20,001–USD 40,000	90	16.9
	USD 40,001–USD 60,000	89	16.7
	≥USD 60,001	166	31.1
BMI category	Under-weight	10	1.9
	Healthy weight	226	42.4
	Over-weight	117	22.0
	Obese	180	33.8
Age group	15–19	35	6.6
	20–24	136	25.5
	25–29	163	30.6
	30–34	130	24.4
	35–39	60	11.3
	≥40	9	1.7
Race/ethnicity	Non-Hispanic White	290	54.4
	Non-Hispanic Black	184	34.5
	Hispanic	59	11.1

Note: Percentages are based on the complete-case sample (N = 533). Detailed Qn21 barrier-item frequencies are listed in Appendix A Table A1.

**Table 3:** Multivariable logistic regression results predicting maternal health status.

Predictor	OR	95% CI	*p*
Smoking status			
Smoker (vs. non-smoker)	0.72	[0.50, 1.03]	0.074
Delay barriers			
Structural barriers (score)	1.09	[0.63, 1.88]	0.767
System barriers (score)	1.77	[1.00, 3.13]	0.047
Informational barriers (θ<sub>info</sub>)	2.42	[1.71, 3.57]	<0.001
Age band (ref = 15–24 y)			
25–34 y	1.05	[0.68, 1.63]	0.819
≥35 y	0.84	[0.51, 1.40]	0.503
Race/Ethnicity (ref = Non-Hispanic White)			
Non-Hispanic Black	1.28	[0.86, 1.90]	0.220
Hispanic/Other	1.26	[0.70, 2.27]	0.446
Income band (ref = ≤USD 20k)			
USD 20k–40k	0.79	[0.49, 1.27]	0.332
>USD 40k	0.83	[0.56, 1.23]	0.349
BMI category (ref = Healthy weight)			
Overweight	1.11	[0.71, 1.74]	0.642
Obese	1.08	[0.72, 1.62]	0.707

Note. OR = odds ratio; CI = confidence interval. Reference categories: age 15–24 y; non-Hispanic White; household income ≤ USD 20k; healthy BMI. θ<sub>info</sub> represents the score for informational barriers in accessing maternal healthcare. Model calibration: Hosmer–Lemeshow *χ2* = 6.5, *df* = 8, *p* = 0.61; Nagelkerke R2 = 0.23.

## Data Availability

Due to the existing data use agreement with the state of Alabama, the entire dataset cannot be shared. However, data supporting the study findings can be made available upon reasonable request.

## Abbreviations

ACME	Average Causal Mediation Effect
ADE	Average Direct Effect
ADPH	Alabama Department of Public Health
BMI	Body Mass Index
CFI	Comparative Fit Index
CI	Confidence Interval
IRB	Institutional Review Board
IRT	Item Response Theory
MDS	Multidimensional Scaling
PNC	Prenatal Care
PRAMS	Pregnancy Risk Assessment Monitoring System
Qn21a	Alabama PRAMS Phase 8 Questionnaire #21a
Qn21b	Alabama PRAMS Phase 8 Questionnaire #21b
Qn21c	Alabama PRAMS Phase 8 Questionnaire #21c
Qn21d	Alabama PRAMS Phase 8 Questionnaire #21d
Qn21e	Alabama PRAMS Phase 8 Questionnaire #21e
Qn21f	Alabama PRAMS Phase 8 Questionnaire #21f
Qn21g	Alabama PRAMS Phase 8 Questionnaire #21g
Qn21h	Alabama PRAMS Phase 8 Questionnaire #21h
Qn21i	Alabama PRAMS Phase 8 Questionnaire #21i
Qn21j	Alabama PRAMS Phase 8 Questionnaire #21j
Qn21k	Alabama PRAMS Phase 8 Questionnaire #21k
RMSEA	Root Mean Square Error of Approximation
SE	Standard Error
SEM	Structural Equation Model
TLI	Tucker—Lewis Index

## Appendix A

**Table A1:** Qn21 Prenatal-care barrier items (frequencies and percentages).

PRAMS Item (Qn21)	No	Yes	% Yes
Couldn’t get an appointment (a)	304	229	43.0
Didn’t have enough money/insurance (b)	356	177	33.2
No transportation (c)	468	65	12.2
Insurance/plan would not start care early (d)	403	130	24.4
Too many other things going on (e)	441	92	17.3
Couldn’t take time off work/school (f)	482	51	9.6
No Medicaid card (g)	387	146	27.4
No childcare (h)	500	33	6.2
Didn’t know I was pregnant (i)	371	162	30.4
Didn’t want anyone to know I was pregnant (j)	480	53	9.9
Didn’t want prenatal care (k)	512	21	3.9

Note: Percentages reflect the same N = 533 analytic sample.

**Table A2:** Fully labeled Qn21 items in Table 1.

Barrier Domain	PRAMS Item Code	Question Text
Delay 1: Informational	Qn21i_Unaware	I didn’t know that I was pregnant
	Qn21j_Secret	I didn’t want anyone else to know I was pregnant
	Qn21k_NoPNC	I didn’t want prenatal care
Delay 2: Structural	Qn21b_Mone	I didn’t have enough money or insurance to pay for my visit
	Qn21c_Transport	I didn’t have any transportation to get to the clinic or doctor’s office
	Qn21e_Busy	I had too many other things going on
	Qn21f_WorkSchool	I couldn’t take time off from work or school
	Qn21h_Childcare	I didn’t have anyone to take care of my children
Delay 3: System	Qn21a_Appoint	I couldn’t get an appointment when I wanted one
	Qn21d_HealthPlan	The doctor or my health insurance plan would not start care as early as I wanted
	Qn21g_Medicaid	I didn’t have my Medicaid card

Note: Qn21 items were derived from the PRAMS Phase 8 dataset provided by the Alabama Department of Public Health. These items represent barriers to prenatal care categorized into structural, informational, and system domains based on the Three Delays Framework. Items were labeled for interpretability and linked directly to the survey questions.

## Appendix B

**Table A3:** Baseline versus adjusted odds ratio for prenatal smoking.

Model	OR	95% CI	*p*
Baseline +	0.83	[0.58, 1.17]	0.28
Adjusted ‡	0.72	[0.50, 1.03]	0.07

Note: Baseline model (marked with +) includes smoking status plus age band, race/ethnicity, income band, and BMI. Adjusted model (marked with ‡) additionally includes informational, structural, and system barrier scores. OR = odds ratio; CI = confidence interval.

**Table A4:** Exploratory mediation analysis (indirect effect of smoking via informational barriers).

Effect	Estimate	95% CI	*p*-Value
ACME (Average)	0.03	[0.01, 0.05]	0.006 ***
ADE (Average)	−0.08	[−0.16, 0.00]	0.056
Total Effect	−0.05	[−0.13, 0.04]	0.232
Prop. Mediated (Average)	−0.44	[−4.62, 4.34]	0.238

Note. ACME = Average Causal Mediation Effect; ADE = Average Direct Effect. Asterisks indicate significance levels: *** *p* < 0.001.

**Table A5:** Exploratory structural-equation model.

Parameter	Estimate	Std. Error	*z*-Value	*p*-Value	95% CI
Mediator Equation					
Smoker_status (a)	0.143	0.058	2.453	0.014	[0.029, 0.257]
Outcome Equation					
Theta_info (d)	0.530	0.079	6.683	<0.001	[0.375, 0.685]
Smoker_status (c′)	–0.199	0.111	–1.797	0.072	[–0.417, 0.019]
Defined Effects					
Indirect Effect (a*d)	0.076	0.032	2.363	0.018	[0.013, 0.139]
Total Effect	–0.123	0.112	–1.106	0.269	[–0.343, 0.097]

Note. Confidence intervals are computed as Estimate ± 1.96 × Std. Error. ACME = Average Causal Mediation Effect (indirect effect); ADE = Average Direct Effect. Smoker_status (a) represents the effect of smoking status on the mediator (informational barriers). Theta_info (d) represents the effect of informational barriers on maternal health. Indirect Effect (a*d) represents the mediated effect of smoking status on maternal health through informational barriers. Smoker_status (c′) represents the direct effect of smoking status on maternal health, controlling for informational barriers as mediating factors. The SEM demonstrated an excellent fit (CFI 0.972, TLI 0.965, RMSEA 0.038 [90% CI 0.032–0.044]).
